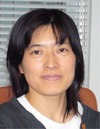# Message From the Editor-in-Chief

**DOI:** 10.2188/jea.JE20150361

**Published:** 2016-01-05

**Authors:** Manami Inoue

Dear Friends and Colleagues,

The official 2014 impact factor for the Journal of Epidemiology was 3.022, placing the journal 32nd among 162 journals in the category of Public, Environmental & Occupational Health and first in the Asia-Pacific region. I sincerely thank all of the editorial team members and reviewers for their outstanding effort in achieving this excellent result. While achieving an impact factor over 3, we have simultaneously faced a rapid increase in the number of submissions. To deal with the considerable number of submissions as promptly and properly as possible, we increased the number of domestic and international associated editors. Our editorial team looks forward to receiving new high-quality submissions from around the world in a broad range of topics in epidemiology, continuing to advance research in all types of health science and policy.

Manami Inoue, MD, PhD
Editor-in-Chief
Journal of Epidemiology 
Project Professor
AXA Department of Health and Human Security
Graduate School of Medicine
The University of Tokyo